# Adaptation and performance of a mobile application for early detection of cutaneous leishmaniasis

**DOI:** 10.1371/journal.pntd.0008989

**Published:** 2021-02-11

**Authors:** Luisa Rubiano, Neal D. E. Alexander, Ruth Mabel Castillo, Álvaro José Martínez, Jonny Alejandro García Luna, Juan David Arango, Leonardo Vargas, Patricia Madriñán, Lina-Rocío Hurtado, Yenifer Orobio, Carlos A. Rojas, Helena del Corral, Andrés Navarro, Nancy Gore Saravia, Eliah Aronoff-Spencer

**Affiliations:** 1 Centro Internacional de Entrenamiento e Investigaciones Médicas, CIDEIM, Cali, Colombia; 2 Universidad Icesi, Cali, Colombia; 3 Grupo i2t, Universidad Icesi, Cali, Colombia; 4 Facultad Nacional de Salud Pública, Universidad de Antioquia, Medellín, Colombia; 5 University of California, San Diego, California, United States of America; Institute of Tropical Medicine, BELGIUM

## Abstract

**Background:**

Detection and management of neglected tropical diseases such as cutaneous leishmaniasis present unmet challenges stemming from their prevalence in remote, rural, resource constrained areas having limited access to health services. These challenges are frequently compounded by armed conflict or illicit extractive industries. The use of mobile health technologies has shown promise in such settings, yet data on outcomes in the field remain scarce.

**Methods:**

We adapted a validated prediction rule for the presumptive diagnosis of CL to create a mobile application for use by community health volunteers. We used human-centered design practices and agile development for app iteration. We tested the application in three rural areas where cutaneous leishmaniasis is endemic and an urban setting where patients seek medical attention in the municipality of Tumaco, Colombia. The application was assessed for usability, sensitivity and inter-rater reliability (kappa) when used by community health volunteers (CHV), health workers and a general practitioner, study physician.

**Results:**

The application was readily used and understood. Among 122 screened cases with cutaneous ulcers, sensitivity to detect parasitologically proven CL was >95%. The proportion of participants with parasitologically confirmed CL was high (88%), precluding evaluation of specificity, and driving a high level of crude agreement between the app and parasitological diagnosis. The chance-adjusted agreement (kappa) varied across the components of the risk score. Time to diagnosis was reduced significantly, from 8 to 4 weeks on average when CHV conducted active case detection using the application, compared to passive case detection by health facility-based personnel.

**Conclusions:**

Translating a validated prediction rule to a mHealth technology has shown the potential to improve the capacity of community health workers and healthcare personnel to provide opportune care, and access to health services for underserved populations. These findings support the use of mHealth tools for NTD research and healthcare.

## Author summary

Cutaneous leishmaniasis (CL) and other tropical diseases primarily affect people in developing and underserved parts of the world where healthcare infrastructure, resources and trained personnel are scarce. Smartphones, connected to the internet and running innovative health-applications—"apps"—have shown promise to bridge gaps in access to healthcare in even the most remote areas. Using participatory and human centered-design methods, we developed a mobile application for Android smartphones that supports community health workers to screen potential cases of CL in rural settings. We developed this application using a previously validated clinical prediction rule and tested it with patients, community workers and health workers in the municipality of Tumaco on the Pacific Coast of Colombia. Results from this study showed that the mobile app was easy to use, could detect cutaneous leishmaniasis in over 95% of cases, and reduced the time to diagnosis by half compared to passive case detection dependent on consultation at referral facilities. Overall, this work showed the value of designing health interventions with early engagement of all users, and supports the general use of mobile health applications for neglected tropical disease research and care.

## Introduction

Cutaneous leishmaniasis (CL) remains a significant public health concern in Colombia due to its high endemicity and prevalence in resource-limited and remote settings, where diagnosis, treatment and monitoring is particularly challenging. In the Americas, Colombia reports the second highest incidence of CL, between 9.99 and 34.17 cases per 100,000 inhabitants over recent years [1]. However, this reported incidence is based on passive surveillance of cases consulting health care institutions, leading to under-ascertainment, -diagnosis and -reporting, which hinder control and the provision of diagnostic and treatment resources in areas of transmission [2].

Diagnosis of CL relies on microscopy or culture of lesion samples, procedures that require highly trained laboratory personnel and/or specialized laboratory equipment, to which there is limited access in areas where transmission occurs [3]. Moreover, many individuals with CL do not seek medical attention because the lesions are painless or because they prefer traditional treatments. Delay or failure to diagnose result in poor outcomes with higher disability, disfigurement and risk of mucosal involvement [4], as well as potentially contributing to ongoing transmission. Access to diagnosis in remote rural areas with limited infrastructure is a priority of control programs yet affordable means of achieving access are not yet available.

Smartphone-based mHealth strategies have recently been developed and evaluated with promising results for surveillance, diagnosis and outcome evaluation of infectious diseases in low-resource settings [5]. Providing mHealth tools to guide community health volunteers (CHV) and health workers (HW) in the presumptive diagnosis of CL in remote areas and their referral for etiologic diagnosis and treatment, could begin to overcome some of the challenges for the control of this disease. Here we describe the user-centered development and field evaluation of a smartphone application that adapted a validated CL prediction rule [3], to provide early, sensitive, case detection and referral for confirmatory diagnosis and treatment in Colombia. In addition to developing the app, this study sought to evaluate its diagnostic performance and usability for detection of CL; and to measure the benefit of its use by CHV, compared to passive case detection, on time to CL diagnosis.

## Methods

### Ethics statement

This study was reviewed, approved and monitored by the institutional ethical review boards of the International Center for Training and Medical Research (CIDEIM) (reference number 1229) and UC San Diego (reference number 160040). During field data collection, the purpose of the study was explained to potential participants. Written consent of participants was obtained from app users, and adults with suggestive lesions. Legal guardians of minors (aged less than 18 years) provided written informed consent. Those minors who were aged at least 7 years provided written informed assent. Cases were sought only in areas considered safe by the CHVs.

### Technology development

We adapted a previously validated clinical prediction rule for CL (Table 1) [3] as a mobile application (app) to support screening for presumptive diagnosis of CL by CHV and HW. The mobile app was created for the Android operating system considering its availability on relatively low-cost smartphones. The app was developed using Android Studio (developer.android.com) and tested using a low-cost, locally available smartphone (Motorola Moto G).

**Table 1:** Components of the clinical prediction rule [3].

Items	Variable	Response	Score*
1	Ulcer with raised borders	No	0
		Yes	1
2	Grouped lesions**	Present	0
		Absent	1
3	Site of lesions	At least one lesion present on the legs, but none on the arms.	1
		No lesions present on the arms or legs (i.e. all lesions are elsewhere).	2
		At least one lesion on the arms.	3
4	Risky activities***	No	0
		Yes	1
5	History of trauma or injury	Yes	0
		No	3
6	Sand fly contact	No	0
		Yes	3

*The item-specific scores are added to obtain the total score. If the total is 7 or greater, then the patient is considered a presumptive CL case and referred to a health provider for etiologic diagnosis and treatment.

**The app defines grouped lesions as those that occur within 10cm and differentiates them from satellite lesions.

***Being in the forest at night, or for >4 hours during the day.

Software development followed principles of human-centered design [6] through an iterative process to achieve an efficient and user friendly app. The user interface was designed collaboratively through a participatory process that included the engineering team, study physicians having expertise in the diagnosis and management of leishmaniasis, and CHV [6]. The design sought to maximize graphic content with an intuitive structure, and to minimize typed data entry and potential inconsistencies. Security features of the app include strong encryption and two-factor authentication.

A second, web-based, application for researchers and physicians was designed to store and process data captured through the mobile application by each end-user (CHV and HW). This web application was developed using a services platform for the collection and management of mobile health data, denoted SND, or System for Neurological Diseases, based on its original purpose (sndweb.azurewebsites.net).

### Study design

We performed a prospective study to evaluate the usability and performance of the mobile application for community-based presumptive diagnosis, and referral for confirmatory diagnosis and treatment.

### Setting

Field evaluation of the mobile app was conducted in one urban area (town of Tumaco) and three rural areas (La Guayacana, Llorente and El Pinde districts) within the municipality of Tumaco (population of 187,084 inhabitants), located on the southwestern Colombian Pacific Coast. The municipality of Tumaco was selected because it has both urban and remote rural communities, and a high burden of CL, with over 300 cases reported per year from 2016 to 2019 [7–10]. Notably, the municipality has a history of internal armed conflict, and electric power is intermittent. The rural part of the municipality has 365 *veredas* (townships), of which only a small proportion have road access. For the rest, the main access is by canoe or launch. In particular, the three rural areas of the current study have limited transportation and access to parasitological diagnosis, which is required for authorization of treatment. Maps facilitated by the principal telecommunication providers show no 3G coverage outside of a few of the larger settlements within the municipality [11–13].

Most cases (approximately 90%) in the Pacific coast region of Colombia are caused by *Leishmania panamensis* with the remainder generally being *L*. *braziliensis*. Other species such as *L*. *mexicana* are rarely encountered. The proportion of these two species of the *Vianna* subgenus can vary by focus and sylvatic or peridomestic transmission.

### Evaluators

Application users lived and worked within communities in the endemic areas of the municipality of Tumaco. The CHV are unpaid community leaders who voluntarily provide health support in rural areas. They include homemakers, teachers, a radio broadcaster and health promoters of the public health system. They provide information about the diagnosis and treatment of CL to members of the community who are either self-identified and seek assistance of the volunteer, or are identified/referred through community members or during gatherings such as social activities, community centers and services, and marketplace encounters as well as in the conduct of their daily activities.

Four CHV were selected from among the leaders of communities in La Guayacana, Llorente and El Pinde who had experience in community work and CL case surveillance. Other app users included two nursing assistants (*auxiliares de enfermería*) and one of the study physicians, who are permanent staff of the CIDEIM clinical facility and CL referral center in the urban center of Tumaco, and two nursing assistants employed by the local health department. CIDEIM (Centro Internacional de Entrenamiento e Investigaciones Médicas) is an independent research institution and led the project. Because of their differing experience in attending leishmaniasis patients, data from the CIDEIM nursing assistants are presented separately from those of the health department, henceforth denoted HW and NA, respectively.

### Enrollment

CHVs employed the mobile application to evaluate individuals with cutaneous lesions by active case finding in their communities. HW and the attending study physician evaluated patients consulting the referral center using the mobile application. The data from the local health department nursing assistants are presented for usability and sensitivity but not for agreement due to the small number of case assessments (n = 5).

### Inclusion criteria for patients

Patients of any gender or race, between 0 and 70 years of age, with at least one cutaneous lesion of less than six months duration, were eligible to participate, and following informed consent, were evaluated with the mobile application.

### Implementation

CHVs and NAs received training on the clinical presentation of CL and all users received training on the conceptual basis of the clinical prediction rule, interpretation of the score obtained after evaluating each of the variables, handling of the smartphone, and use of the application. Refresher training, covering the same topics, was conducted six months after initial training, to review and reinforce use of the tool. Individualized training was also provided as needed. A smartphone (Motorola Moto G3) was provided to each user. CHVs and NAs were instructed to apply the prediction rule using the mobile application and refer all evaluated patients, independently of the score, to the CIDEIM clinic for further medical and parasitological evaluation. Although in standard use, only presumptively diagnosed cases would be referred, for the purposes of the current study, all patients were referred in order to fully evaluate the performance and operating characteristics of the mobile application. All patients evaluated by CHVs and NAs were also evaluated at the CIDEIM clinical facility by one or both HW and the study physician who used the mobile app prior to clinical and parasitological assessment.

At the end of the intervention, users were asked to complete an assessment questionnaire consisting of 11 statements on the use and acceptance of the app. The options for each response were displayed on a Likert scale of 1, 2, 3, 4, or 5.

### Clinical assessment and management

Clinical history and physical examination were conducted by the study physician who also obtained samples for parasitological evaluation. Treatment and additional procedures (biopsies, laboratory tests) were conducted in accordance with the standard of care [14].

### Parasitological assays

Microscopic evaluation for the presence of amastigotes of *Leishmania spp* in tissue smears, and culture of tissue aspirates in biphasic media, were performed as described elsewhere [15]. Diagnosis of CL was confirmed by visualization of amastigotes in tissue smears or by culture of *Leishmania spp* from tissue aspirates.

### Statistical analysis

Data were extracted from the SND platform and clinical histories, and analyzed using R software version 3.6 [16]. Operating characteristics of the mobile application for presumptive diagnosis of CL were determined considering parasitological diagnosis as the gold standard, and were presented with their corresponding binomial confidence intervals, calculated using the Wilson method [17] (‘binom’ package in R). For concordance analysis of between-rater assessment using the application, Cohen’s kappa was used [18–20] (‘irr’ package in R). Kappa is a chance-adjusted measure of concordance, accounting for higher agreement for a characteristic with high prevalence. For concordance between ordered categories, such as the total score, quadratic weighting of differences was used [21]. Kappa was not calculated if two or more of the four cells were zero. The arbitrary, but widely used, nomenclature of Landis and Koch [20] was used to describe the strength of concordance based on the value of kappa. To designate a presumptive case using the application score, the previously validated threshold point was set at 7 (a score of ≥ 7 or < 7) [22]. The *t* test was used to compare mean differences in time from onset of symptoms and diagnostic assessment, between the different types of users of the application.

## Results

### Adaptation of the clinical prediction rule to a mobile application

The clinical prediction rule was iteratively adapted to the form of an application for mobile phones (S1 and S2 Movies and S1 Software (available from: https://play.google.com/store/apps/details?id=guaralrpctwo.cideim.guaral.guaralrpc)). The initial approach was to identify the main components of the application and to design an interaction model that allows the user to focus on the patients rather than on the tool.

**Fig 1:**
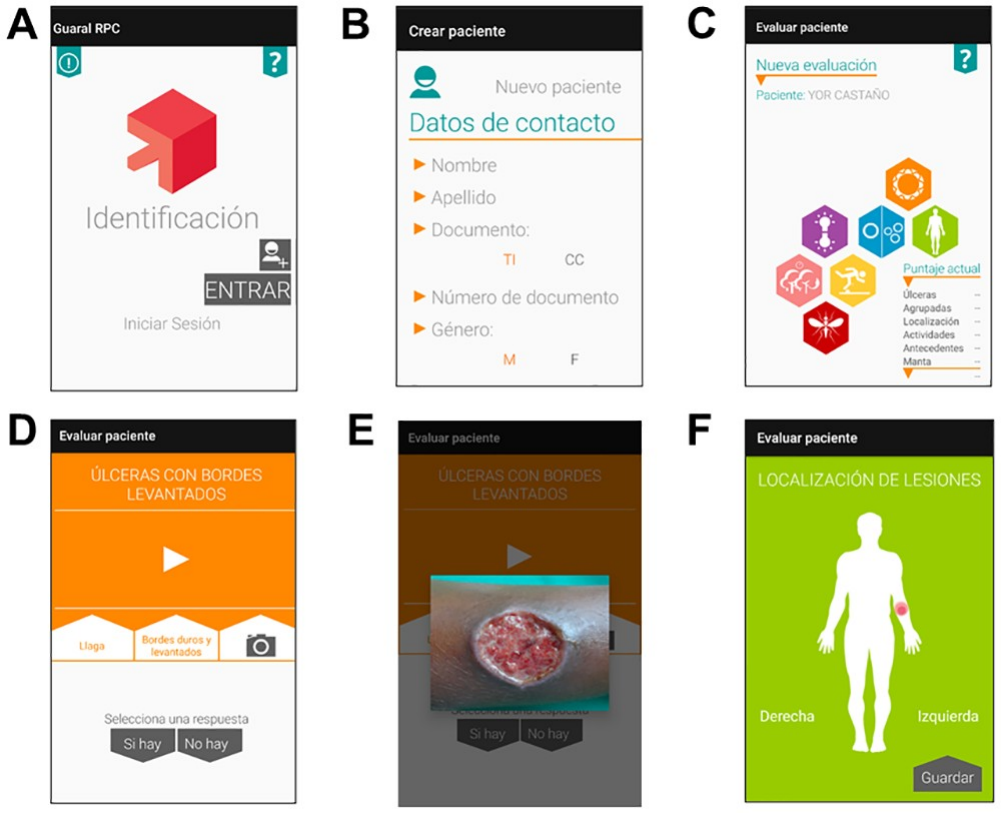
mHealth tool (app) for presumptive diagnosis of leishmaniasis. (A) Homepage requesting user’s name and ID. (B) Personal information for each patient. (C) Main screen with six icons representing the six items of the prediction rule. (D) Menu for Item 1, lesions. (E) Image for user guidance, showing a typical CL lesion. (F) Interface for item 3, allowing the user to select the exact lesion site(s).

After the user logs in with an identifier and password, and enters the basic identification data of the patient (Fig 1A-B), they reach the main screen, which schematically shows six color-coded icons (Fig 1C), each corresponding to one element of the prediction rule (Table 1). The iconography was chosen to facilitate navigation, and guide the question content of the prediction rule app (Fig 1D–1F). Users can tap on icons in any order to enter the corresponding information. Some icons also have multimedia guidance. For instance, the orange lesion icon (Fig 1D) asks about the presence of lesions with raised borders and shows a typical CL ulcer (Fig 1E). Users may indicate the site on the body where each lesion is located simply by touching the screen (Fig 1F). The scoring for this item, lesion site(s) (number 3 in Table 1), is relatively complex and the app obviates the need for manual calculation, which was previously found to be a main source of errors.

**Fig 2:**
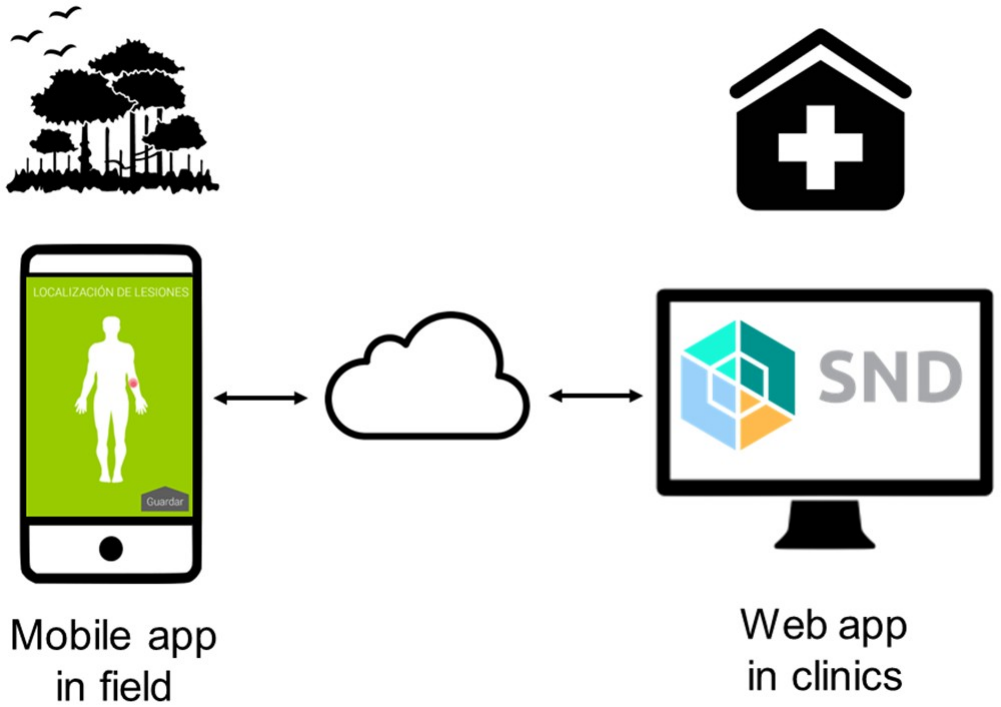
Data flow. Users capture the information in the field via the mobile application and synchronize data when a connection is available, via secure cloud storage. The data can be accessed and analyzed by study physicians or researchers via the desktop web application. Sources: the app screen images are created by the authors; other images are in the public domain, taken from publicdomainvectors.org (cloud) and icon-library.net (others).

The data flow and storage are shown in Fig 2. As necessary, data captured by the mobile application can be stored off-line (locally on the phone) and transmitted later to the SND platform, when connectivity is re-established.

### Usability of the mobile application

**Fig 3:**
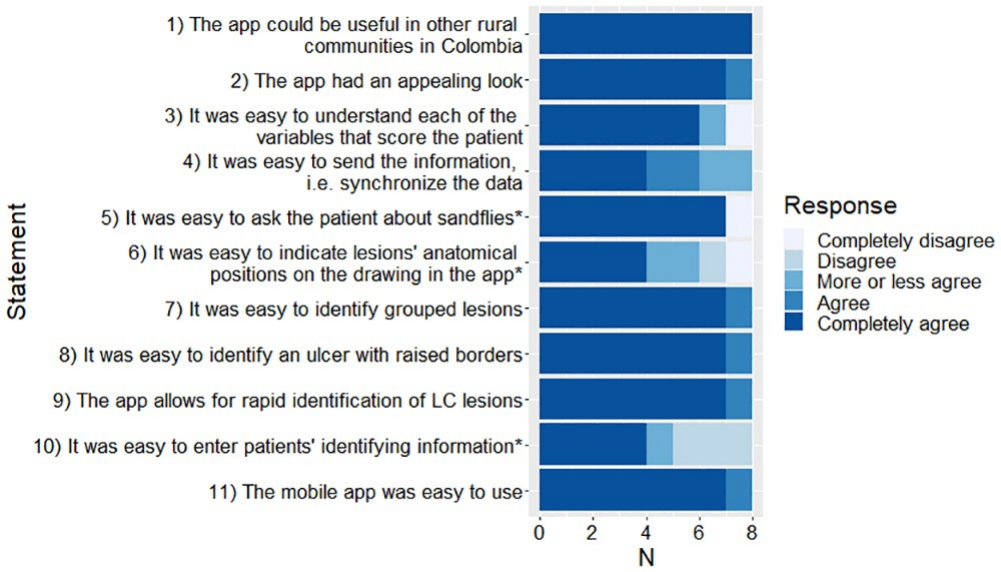
Results of questionnaire on usability. The median (range) score per question are: Q1, 1 (range 1–1); Q2, 1 (1–2); Q3, 1 (1–5); Q4, 1.5 (1–3); Q5, 1 (1–5); Q6, 2 (1–5); Q7, 1 (1–2); Q8, 1 (1–2); Q9, 1 (1–2); Q10, 2 (1–4); Q11, 1 (1–2). *Indicates statements that were posed in terms of difficulty of use, rather than ease of use. Here they have been inverted, in order to facilitate comparability across all the statements.

Eight users (3 CHV, 2 HW, 1 physician and 2 local health department nursing assistants) completed a questionnaire on the usability of the app (Fig 3). The app scored favorably on all statements, with a median score of 1, 1.5 or 2, on a scale of 1–5, 1 being the most favorable.

### Evaluation of patients with lesions suggestive of CL using the mobile app

Between February 2015 and March 2016, 122 participants with lesion(s) suggestive of CL were evaluated by the physician using the mobile app. Of these, 85 were also evaluated by HW1, 96 by HW2, and 15 by one of the four CHV. Demographic and clinical characteristics of the study participants are summarized in Table 2. Patients were predominantly male (81%), of African descent (50%), and between 15 and 44 years of age (73%). During the study period, a further four people were screened by the CHV with the app but were not included due to: lack of parasitological data (n = 2), reported duration of lesions of more than six months, which was an exclusion criterion (n = 1), and missing evidence of consent (n = 1).

**Table 2:** Socio-demographic and clinical characteristic of study participants.

Demographic characteristics of participants (n = 122)	Number (%)
**Age (years)**	
Under 5	4 (3.3)
5–14	17 (13.9)
15–44	89 (73.0)
Over 45	12 (9.8)
**Gender**	
Female	23 (18.9)
Male	99 (81.1)
**Ethnicity**	
Afro descendant	61 (50.0)
Indigenous	31 (25.4)
White	1 (0.8)
Mestizo	29 (23.8)
**Place of residence**	
Rural	112 (91.2)
Periurban	10 (8.2)
**Number of lesions by participant**	
1	74 (60.7)
2	18 (14.8)
3	13 (10.7)
4	3 (2.5)
>4	14 (11.5)
**Duration of lesions (weeks)**	
<4	54 (44.2)
5–9	31 (25.4)
10–14	20 (16.4)
>14	17 (13.9)
Type of lesion*	
Ulcer	227 (89.0)
Plaque	4 (1.6)
Papule	11 (4.3)
Scar	3 (1.2)
Scar with active borders	10 (3.9)
Location of lesion*	
Head and neck	41 (16.1)
Trunk	45 (17.6)
Upper limbs	113 (44.3)
Lower limbs	56 (22.0)
**Parasitological diagnosis**	
Positive	107 (87.7)
Negative	14 (11.5)
Indeterminate (Etiology not determined)	1 (0.8)

*Total of 255 lesions, due to some patients having multiple lesions.

Of the 122 participants, most (n = 107, 88%) had parasitologically confirmed CL (Table 2). Of those positive, all but one were positive on microscopy, with the exception being positive on culture. Microscopy was done for all those parasitologically positive, and culture on 22% (24/107). Among those who were parasitologically negative (n = 14, 11%), other diagnoses were clinically defined by the attending physician and included predominantly bacterial infections and vascular ulcers, as well as one case each of sporotrichosis and squamous cell carcinoma. Most had a single lesion (61% of 122) and a large majority (89%) presented ulcerated lesions, with 44% located on exposed areas like the arms. More than half of the patients reported having a lesion with >1 month duration.

### Diagnostic performance

The sensitivity of the mobile app was very high (>95%) for all users (Table 3). The CHV results are aggregated because no potential CL patient was assessed by more than one of them. Specificity was not determined due to the small number of participating patients having a confirmed diagnosis of another etiology.

**Table 3:** Sensitivity of the clinical prediction rule for presumptive diagnosis of cutaneous leishmaniasis, relative to parasitological diagnosis.

App user(s)	Parasito-logical diagnosis*	Presumptively CL-negative (score <7)	Presumptive CL case (score ≥7)	Total	Sensitivity (95% confidence interval)
Physician					
	Negative	1	13	14	
	Positive	3	103	106	97.2% (92.0–99.0%)
Health Worker 1					
	Negative	1	8	9	
	Positive	0	76	76	100% (95.2–100%)
Health Worker 2					
	Negative	1	8	9	
	Positive	0	86	86	100% (95.7–100%)
Four Community Health Volunteers					
	Negative	1	4	5	
	Positive	0	10	10	100% (72.2–100%)
Two nursing assistants					
	Negative	0	0	0	
	Positive	0	5	5	100% (56.6–100%)

*Omitting the one indeterminate result. Also, none of the users examined all the potential cases, so each set of rows has a total less than the total in Table 2.

### Inter-observer agreement of the mobile application

Overall, with the threshold score of seven for presumptive diagnosis of CL using the mobile application, high agreement was observed between the physician and the HW and CHV (Table 4). However, the concordance (kappa) was low. For example, comparing the assessment of HW1 with that of the physician, there were 80 agreements on being a presumptive case, but no agreements on being CL-negative, yielding a kappa of -0.02. The agreement for individual components of the score varied from ‘almost perfect’ [20] for the site of the lesions, to ‘slight’ or ‘poor’, for the item grouped lesions.

**Table 4:** Concordance between the evaluation performed by the CHV and the HW compared to the evaluation performed by the physician using the mobile application.

	Health Worker 1 (85 evaluations)		Health Worker 2 (96 evaluations^¶^)		Four Community Health Volunteers (15 evaluations)	
	Agreement	Kappa (95% CI)	Agreement	Kappa (95% CI)	Agreement	Kappa (95% CI)
Presumptive diagnosis (CL case or not)	95%	-0.02 (-0.20, 0.17)	98%	-0.01 (-0.21, 0.19)	93%	*
Scored on the app (range 1–12)	81%	0.26 (0.05, 0.48)	82%	0.19 (-0.01, 0.39)	73%	-0.05 (-0.55, 0.45)
Individual components of the score						
Ulcer with raised borders	91%	0.31 (0.15, 0.46)	95%	*	87%	0.44 (0.02–0.87)
Grouped lesions	76%	-0.04 (-0.16, 0.07)	68%	0.07 (-0.08, 0.23)	53%	0.09 (-0.38, 0.55)
Site of lesions	89%	0.90 (0.69, 1^§^)	95%	0.96 (0.76, 1^§^)	93%	0.95 (0.45, 1^§^)
Risky activities	99%	0.79 (0.59, 1^§^)	98%	0.66 (0.46, 0.86)	93%	*
History of trauma or injury	96%	*	100%	*	100%	*
Sand fly contact	99%	0.66 (0.46, 0.86)	98%	0.49 (0.32, 0.66)	93%	0.76 (0.27, 1^§^)

^¶^One more than in Table 3 because this person evaluated the parasitologically indeterminate patient who was excluded from Table 3.

*Not calculated because of small sample size (see Methods).

^§^Upper confidence limit truncated at 1.

### Impact of active community case finding and referral versus facility-based passive detection using the mobile app

Individuals identified and evaluated within their communities using the mobile app by CHV were diagnosed on average 4.4 weeks (n = 15, range 2–12) after initiation of symptoms, compared to 8.5 weeks (n = 102, range 2–21) for those directly consulting the CIDEIM referral center, where they were evaluated by the health personnel using the app. Hence the difference was 4.1 weeks, 95% confidence interval 1.3–6.8, p = 0.005.

## Discussion

This study reports the development and evaluation of a mobile adaptation of a validated clinical prediction rule [3,6] for presumptive diagnosis of CL by CHV and health workers. Our findings join a growing corpus of literature demonstrating that mHealth systems using mobile telephones have the potential to be viable and cost-effective strategies to improve access to healthcare, opportune diagnosis and epidemiological surveillance for under-served populations [23]. The current study was carried out in a resource-constrained region with limited health care infrastructure and access to diagnosis and treatment of CL. Furthermore, ongoing conflict, a circumstance that is often associated with CL [24] is a contributing factor to under-reporting and under-ascertainment of this disease.

In this challenging context the training process of the CHVs was re-enforced by their frequent contact with the population at risk of CL. Their adoption of the mHealth tool was facilitated by their ready recognition of the risk factors evaluated (S1 Movie).

The app performed with high sensitivity (>95%) in detecting true CL cases, whether in the hands of a physician, health worker, or community health volunteers. The results of this study confirmed the discriminatory cutoff score of ≥ 7 for presumptive CL derived from the prior validation study. These findings support the feasibility of screening with the app as a first step to triage patients for the confirmatory parasitological diagnosis required to authorize and administer treatment. The app could economize the use of rapid diagnostic (confirmatory) tests by CHV when such tests become available. Importantly, besides facilitating active case detection, the app encourages and empowers community health volunteers through its interactive and intuitive format, and access to the support of health professionals.

Compared to the previous validation study that was based on a mechanical prediction rule device [3], a higher percentage of agreement was observed with the application for most of the prediction rule variables for HW and CHV when they were compared with the study physician. This improvement is likely due to the instructive information that is provided by the application, which facilitates the understanding and assessment of the variables. This improvement was generally observed both for variables related to the clinical characteristics of the lesion (ulcer with raised borders, and lesion site), and for variables related to clinical history such as the presence of risk activity when the infection was acquired and lesion developed. The exception was the variable “no grouped lesions”, which had a lower level of agreement with the application compared to the mechanical prediction rule. This is not surprising because it has been one of the most difficult variables to evaluate by non-medical personnel, since the development of the prediction rule in 1993 [22]. The adaptation that was made for the application evidently did not overcome the limitation that we have experienced with this variable. However, this contrasts with the results of the usability assessment (Fig 3), in which most of the user participants agreed about the ability of the application to identify grouped lesions. The characteristics of lesions may differ in other regions, e.g. in terms of raised borders and whether nodular or ulcerated, and the app could require adaptation in those terms to be effective elsewhere.

In this study we had limited ability to assess other operating characteristics of the app (in particular, specificity and negative predictive value), because few of the eligible cases were of an etiology other than CL. In this area, common differential diagnoses are vascular ulcers, squamous cell carcinomas and bacterial infections.

Inter-observer agreement—the percentage of assessments with the same result—was high, although this was again driven by the high proportion of true CL cases among the participants. We hope to address this limitation in further studies of the app which are currently underway. In areas of low prevalence, the specificity, and positive and negative predictive values of the app score are critical, and it is important to train users in epidemiological, clinical, diagnostic, dermatologic concepts of leishmaniasis, and differential diagnosis.

The point estimates of the chance-corrected concordance (kappa) for the individual components of the score ranged from very high to zero (for grouped lesions), and were generally lower than found in a previous study using a mechanical version of the prediction rule that included a higher proportion of etiologies different from CL [3], although the confidence intervals were generally wide. Another limitation of our study is that most of the data are from users with a higher level of education, and experience with CL, than the target population of users (i.e. CHV). A major reason for this was security concerns, since those perceived as social activists are often targeted by outlaw groups.

The availability of the app as a tool for active case detection in the hands of CHV in rural areas resulted in a significantly shorter interval between the onset of symptoms and diagnosis as compared to the current practice of passive detection through patient consultation of health facilities and health personnel in urban Tumaco. Active case detection in the community, together with referral, has the potential to promote prompt parasitological diagnosis and treatment as well as improve case ascertainment and reporting. Although the operating characteristics of the app require further evaluation, particularly in relation with dermal lesions of different etiologies, the mHealth app could become an integral part of an active community-based surveillance model in low resource areas, possibly involving schools.

The didactic components of the app contribute to its potential as a training resource for CL detection by health care personnel. Future increase in 3G coverage in endemic areas of Colombia anticipate a scenario where mHealth will be even more effective. In the meantime, portable antennas could improve access, while awareness could be enhanced via broadcast media such as radio. On the other hand, although each individual’s scores are necessary for a research study such as the current one, in routine use it may not be necessary to transmit the scores to a physician once they have been generated by the app.

The participation of community or primary health workers presumptively diagnosing cutaneous leishmaniasis based on clinical diagnostic or clinical-historical prediction models, has been evaluated in other settings [25,26]. Another study conducted in Colombia, in three municipalities of the Department of Santander, also found that CHW achieved good sensitivity when they sought a presumptive clinical diagnosis of CL, using a qualitative diagnostic Likert five level-scale [26].

Demonstration of the sensitivity and feasibility of surveillance for active case detection at the community level using mHealth technology not only supports its potential to promote opportune diagnosis and treatment, but also of determining the true magnitude of this neglected disease at the local and national levels in Colombia, as well as potentially in other South American countries. This knowledge would inform and support the allocation of resources for prevention and control.

## Supporting information

S1 Movie Guaral app overview movie, file name “Guaral app overview movie 270px.mp4”. Overview movie of the overall study using the mobile application. A higher resolution version can be accessed at: https://www.youtube.com/watch?v=DDJ08yesYOM&feature=youtu.be.
(MP4)

S2 Movie Guaral app tutorial video, file name “VIDEO TUTORIAL GUARAL APP INGLES SD.mp4”. Tutorial video showing how to use the mobile application. A higher resolution version can be accessed at: https://www.youtube.com/watch?v=OjRMA2G92ik&index=9&list=UUYkYVZC4pGQDYPaYe73rRww.
(MP4)
